# Colorectal surveillance outcomes from an institutional longitudinal cohort of lynch syndrome carriers

**DOI:** 10.3389/fonc.2023.1146825

**Published:** 2023-04-24

**Authors:** Gabriel del Carmen, Laura Reyes-Uribe, Daniel Goyco, Kyera Evans, Charles M. Bowen, Jennifer L. Kinnison, Valerie O. Sepeda, Diane M. Weber, Julie Moskowitz, Maureen E. Mork, Selvi Thirumurthi, Patrick M. Lynch, Miguel A. Rodriguez-Bigas, Melissa W. Taggart, Y. Nancy You, Eduardo Vilar

**Affiliations:** ^1^ Department of Clinical Cancer Prevention, The University of Texas MD Anderson Cancer Center, Houston, TX, United States; ^2^ McGovern Medical School, University of Texas Health Science Center, Houston, TX, United States; ^3^ Department of Medicine, Baylor College of Medicine, Houston, TX, United States; ^4^ Clinical Cancer Genetics Program, The University of Texas MD Anderson Cancer Center, Houston, TX, United States; ^5^ Department of Gastroenterology, Hepatology and Nutrition, The University of Texas MD Anderson Cancer Center, Houston, TX, United States; ^6^ Department of Colorectal Surgery, The University of Texas MD Anderson Cancer Center, Houston, TX, United States; ^7^ Department of Pathology, The University of Texas MD Anderson Cancer Center, Houston, TX, United States

**Keywords:** lynch, colorectal < cancer type, surveillance, colonoscopy, premalignancies

## Abstract

**Objective:**

Lynch Syndrome (LS) carriers have a significantly increased risk of developing colorectal cancer (CRC) during their lifetimes. Further stratification of this patient population may help in identifying additional risk factors that predispose to colorectal carcinogenesis. In most LS patients CRC may arise from adenomas, although an alternative non-polypoid carcinogenesis pathway has been proposed for *PMS2* carriers. Using data from our institutional LS cohort, our aim was to describe our current colorectal screening outcomes with a focus on the incidence of adenomas in the context of different MMR genotypes and patient demographics such as gender, race, and ethnicity.

**Design:**

We collected demographics, genetic, colonoscopy, and pathology results from a total of 163 LS carriers who obtained regular screening care at MD Anderson Cancer Center. Data were extracted from the electronic health records into a REDCap database for analysis. Logistic regressions were performed to measure the association between MMR variants and the likelihood of adenomas, advanced adenomas, and CRC. Then, we analyzed the cumulative incidences of these outcomes for the first 36 months following enrollment using Kaplan-Meier incidence curves, and Cox proportional hazard regressions.

**Results:**

On multivariate analysis, age (≥45 years old) was associated with an increased risk of developing adenomas (*P=*0.034). Patients with a prior or active cancer status were less likely to develop adenomas (*P*=0.015), despite of the lack of association between surgical history with this outcome (*P*=0.868). We found no statistically significant difference in likelihood of adenoma development between *MLH1* and *MSH2/EPCAM, MSH6, and PMS2* carriers. Moreover, we observed no statistically significant difference in the likelihood of advanced adenomas or CRC for any measured covariates. On Cox proportional hazard, compared to *MLH1* carriers, the incidence of adenomas was highest among *MSH2/EPCAM* carriers during for the first 36-months of follow-up (*P*<0.001). We observed a non-statistically significant trend for Hispanics having a higher and earlier cumulative incidence of adenomas compared to non-Hispanics (*P=*0.073). No MMR carrier was more likely to develop advanced adenomas. No difference in the incidence of CRC by MMR gene (*P=*0.198).

**Conclusion:**

Screening recommendations for CRC in LS patients should be based on specific MMR variants and should also be tailored to consider patient demographics.

## Introduction

Lynch syndrome (LS) is a genetic condition associated with an increased risk of developing multiple types of cancers and it is best known for being the most frequent cause of inherited colorectal and endometrial cancers (1, 2). Colorectal cancer (CRC) is the third most common cancer by incidence worldwide and the second by mortality among all cancers (3). While the general population has an approximately 5% life-time cumulative risk of developing CRC, LS carriers have estimated risks between 10% and 50% depending on the mismatch repair (MMR) gene (4–6). LS carcinogenesis is secondary to alterations in the mismatch repair (MMR) system, which corrects base-pairing errors that occur during DNA replication (7). More specifically, LS results from constitutional variations in one of the four MMR genes (*MLH1*, *MSH2*, *MSH6*, and *PMS2*) or deletion within *EPCAM*, which promotes hypermethylation and silences *MSH2* (7). Given the heightened risk of the development of CRC for this patient population, adequate colonoscopy screening intervals are crucial for the identification and subsequent removal of pre-cancers (i.e., adenomas). Projected yearly transition rates from advanced adenomas to carcinomas range between 2.6 to 5.6% in the general population, with age being the most significant risk factor (8, 9). The transition rate is estimated to be even higher in the LS cohort, although the true transition rate is unknown; therefore, this population requires more frequent colonoscopies (10) with current recommendations on the age to start screening and frequency intervals based on specific gene variants. In fact, *MLH1, MSH2*, and *EPCAM* carriers are advised to initiate screening at age 20-25 or, if diagnosed before age 25, 2-5 years before the earliest diagnosis of CRC in the family, with intervals every 1-2 years (11–13). In contrast, screening should start later and be performed less frequently among *MSH6* and *PMS2* carriers with the first colonoscopy at age 30-35 or, if diagnosed before age 30, 2-5 years prior to a familial CRC diagnosis, with intervals every 1-3 years (11). With a focus on gene variants, the role of patient demographics, particularly the contribution of race and ethnicity, has not been appropriately addressed in the current recommendations. This omission might be significant since it has already been documented that racial and ethnic minorities are often referred for genetic testing at a diminished rate despite universal screening and genetic testing recommendations (14). Therefore, there is an unmet need to optimize CRC screening in this specific population of patients.

Here, we report the colonoscopy findings from a cohort of LS participants from a single institution. This longitudinal dataset allow us to characterize and report colorectal screening outcomes. In making this information available, our goal is to stimulate a re-evaluation of current cancer surveillance recommendations as well as to contribute to an understanding of how patient demographics affect colorectal adenoma and CRC development in the LS population.

## Materials and methods

### Study design and participants

A total of 163 LS patients were recruited to an IRB-approved protocol (MDACC IRB# PA12-0327) between March 2013 and March 2020 at the University of Texas MD Anderson Cancer Center (MDACC). Eligible participants were 18 years or older at the time of enrollment and were either proven to be carriers or obligate carriers of a pathogenic or likely-pathogenic variant in one of the four MMR genes (*MLH1*, *MSH2*, *MSH6*, or *PMS2*) or *EPCAM*. Patients underwent colonoscopy/flexible sigmoidoscopy per standard of care indications. After patient consent, we retrospectively and prospectively collected individual patient data with manual review from the electronic medical records in a REDCap database. Data on hospital visits, colonoscopy results, and past surgeries were collected for analysis. Loss to follow-up (LTFU) was defined as the absence of patient contact with clinic or procedure visits for more than 36 months at the latest captured point in data collection (censored in March of 2020).

### Aims

The primary endpoints of this study were to (1): identify the degree of association between MMR genetic variation and the development of colorectal adenomas, advanced adenomas, and CRCs in our LS institutional cohort; and (2) determine the association of demographic characteristics with the development of colonoscopy findings during follow-up.

### Statistical analysis

Patient demographics were summarized by descriptive statistics including surgical history (**Table 1**). The category of ‘other surgeries’ included the following procedures: subtotal colectomy with ileorectal anastomosis, abdominoperineal resection, low anterior resection, and partial colectomy. ‘Small bowel surgeries’ included small bowel resections with enterostomy and Billroth II gastrojejunostomies. Data on ‘adenomas’ as a whole included tubular adenomas, sessile serrated adenomas, and tubulovillous adenomas. Therefore, we did not incorporate hyperplastic or inflammatory polyps in outcome assessment. Advanced adenomas were defined as adenomas ≥ 1 centimeter in diameter, presenting villous features, and/or presence of high-grade dysplasia (HGD). Mean number adenomas per procedure (MAP) was calculated as the number of all adenomas from colonoscopies over the number of all colonoscopies performed. The mean number of adenomas per positive procedure (MPP+) was calculated as the number of all adenomas from colonoscopies over the number of all colonoscopies with adenomas found.

**Table 1:** Patient demographics.

Demographics	(N=163)
Age at Enrollment	
18-29	20 (12.3%)
30-39	29 (17.8%)
40-49	31 (19.0%)
50-59	42 (25.8%)
60-69	29 (17.8%)
70-79	10 (6.1%)
>80	1 (0.6%)
Sex	
Female	95 (58.3%)
Male	68 (41.7%)
Race	
White or Caucasian	128 (78.5%)
Black or African American	6 (3.7%)
Asian	7 (4.3%)
American Indian	1 (0.6%)
Unknown Race/Ethnicity	1 (0.6%)
Other	20 (12.3%)
Ethnicity	
Hispanic or Latino	22 (13.5%)
Not Hispanic or Latino	134 (82.2%)
Unknown	7 (4.3%)
Cancer History (n)*	113 (69.3%)
Colon	68 (60.2%)
Rectum	14 (12.4%)
Small Bowel	1 (0.9%)
Urothelial Tract	5 (4.4%)
Endometrial	23 (20.4%)
Other	43 (38.1%)
Genes	
*MLH1*	54 (33.1%)
*MSH2/EPCAM/TACSTD1*	60 (36.8%)
*MSH6*	33 (20.2%)
*PMS2*	16 (9.8%)
Colorectal surgery	71 (43.6%)
Right Hemicolectomy	36 (50.7%)
Total Colectomy with Ileostomy	1 (1.4%)
Total Colectomy with Ileorectal Anastomosis	4 (5.6%)
Left Hemicolectomy	6 (8.5%)
Sigmoid Colon Resection	5 (7.0%)
Other Surgeries	16 (22.5%)
Small Bowel Resection	3 (4.2%)

*, Note that patients can present with more than one cancer type.

We conducted two separate analyses in this study. The first one evaluated LS carrier characteristics by MMR gene for the entirety of our study period (2013-2020 and inclusive of a retrospective review of the data prior to 2013 in a fraction of the participants). Carrier characteristics were summarized by MMR gene (**Table 2**), and significance of these MMR variant characteristics was determined using Pearson’s chi-squared (χ²) test for categorical variables and analysis of variance (ANOVA) for continuous variables. Furthermore, multivariate analyses were performed for adenomas, advanced adenomas, and CRC. These analyses were controlled for MMR gene variation, age, gender, ethnicity, race, smoking status, surgical history, and cancer status (defined as previvor compared to active cancer and survivor) over the entire study period. The results were reported as odds ratios (ORs) using logistic regressions. In the second one, we evaluated the time to our outcome(s) of interest for each patient for a 36-month period, which was the minimum period of follow-up for all participants in our cohort, using Kaplan-Meier cumulative incidence curves. These curves provided a descriptive overview of the time to incidence of adenoma, advanced adenoma, and CRC during the 36-month follow-up. We determined these incidences by MMR gene, ethnicity, and gender. Carriers were considered at-risk from the point they entered our cohort until they developed the outcome of interest (i.e., adenoma, advanced adenoma, or CRC). At the time of outcome development, patients were no longer considered at-risk and were not included in the analysis for the subsequent months. As some patients were found to have developed their first adenoma, advanced adenoma, or CRC at the time of enrollment, they were not included within the Kaplan-Meier cumulative incidence analysis. Death was included as a competing risk in our analysis, though no patients died prior to developing the outcomes of interest. For Kaplan-Meier cumulative incidence curves, statistical significance was determined using a Cox proportional regression analysis with Breslow method, and the results were reported as hazard ratios (HRs). For all analyses, a *P*-value≤0.05 was considered statistically significant. All data were analyzed using STATA v16.0 (STATA Corp., TX, US).

**Table 2:** LS Carrier Characteristics by MMR Gene.

	MLH1	MSH2/EPCAM	MSH6	PMS2	Total	P-Value
**N**	54 (33.1%)	60 (36.8%)	33 (20.2%)	16 (9.8%)	163	
**Status at Enrollment**						0.154
Previvor	19 (35.9%)	22 (37.3%)	13 (40.6%)	6 (37.5%)	60 (37.0%)	
Survivor	18 (34.0%)	23 (39.0%)	10 (31.3%)	9 (56.3%)	61 (37.7%)	
Active Cancer	16 (30.2%)	14 (23.7%)	9 (28.13)	1 (6.3%)	41 (25.3%)	
**Age at enrollment**	42.6 (IQR, 34-50; range, 18-73)	44.5 (IQR, 33-53; range, 18-77)	51.9 (IQR, 44-61; range, 20-71)	46.5 (IQR, 33-59; range, 24-72)	46.0 (IQR, 35-56; range, 18-77)	0.082
**Mean Interval Between Follow-Up (Months)**	12.5 (IQR, 0-42; range, 0-128)	14.2 (IQR, 0-50; range, 0-118)	12.5 (IQR, 0-35; range, 0-59)	10.5 (IQR, 2-13; range, 1-31)	13.1 (IQR, 0-59; range, 0-128)	0.188
**Mean Total Follow-Up Period (Years)**	9.2 (IQR, 0.4-22.0; range, 0-22.0)	9.3 (IQR, 0.3-21.3; range, 0-21.3)	7.3 (IQR, 1.2-14.4; range, 0.6-14.4)	4.4 (IQR, 1.1-8.8; range, 0.1-8.8)	8.7 (IQR, 0-22.0; range, 0-22.0)	<0.001
**Sex**						0.336
Female	23 (43.4%)	37 (61.7%)	21 (63.6%)	11 (68.8%)	95 (58.3%)	
Male	30 (56.6%)	23 (38.3%)	12 (36.4%)	5 (31.3%)	68 (41.7%)	
**Race**						0.555
White or Caucasian	37 (69.8%)	47 (79.7%)	28 (87.5%)	13 (81.3%)	127 (78.4%)	
Black or African American	2 (3.8%)	1 (1.7%)	2 (6.3%)	1 (6.3%)	6 (3.7%)	
Asian	5 (9.4%)	1 (1.7%)	1 (3.1%)	0 (0.0%)	7 (4.3%)	
American Indian	1 (1.9%)	0 (0.0%)	0 (0.0%)	0 (0.0%)	1 (0.6%)	
Unknown Race	0 (0%)	9 (15.3%)	0 (0.0%)	0 (0.0%)	20 (12.4%)	
Other	8 (15.1%)	1 (1.7%)	1 (3.1%)	2 (12.5%)	1 (0.6%)	
**Ethnicity**						0.479
Hispanic	10 (18.5%)	9 (15.3%)	1 (3.1%)	2 (12.5%)	22 (13.5%)	
Non-Hispanic	41 (75.9%)	48 (81.4%)	29 (90.6%)	14 (87.5%)	134 (82.2%)	
Unknown	3 (5.6%)	2 (3.4%)	2 (6.3%)	0 (0%)	7 (4.3%)	
First Colonoscopy						
**Mean Age (years)**	43.7 (IQR, 36-50; range, 25-73)	43.8 (IQR, 36-52; range, 18-72)	51.4 (IQR, 42-61; range, 20-71)	49.5 (IQR, 32-59; range, 24-72)	45.8 (IQR, 37-55; range, 18-73)	0.043
**Adenoma Count**						0.959
0	21 (58.3%)	19 (48.7%)	8 (44.4%)	6 (66.7%)	54 (53.5%)	
1	6 (16.7%)	13 (33.3%)	6 (33.3%)	2 (22.2%)	27 (26.7%)	
2	3 (8.3%)	1 (2.6%)	2 (11.1%)	1 (11.1%)	7 (6.9%)	
≥3	1 (2.8%)	2 (5.1%)	1 (5.6%)	0 (0.0%)	4 4.0%)	
**Advanced Adenoma Count**	2 (5.6%)	2 (5.1%)	1 (5.6%)	0 (0.0%)	4 (4.0%)	0.479
**CRC Count**	3 (8.3%)	2 (5.1%)	0 (0.0%)	0 (0.0%)	5 (5.0%)	0.667
Screening and Colonoscopy Results						
**Number of Colonoscopies**	184 (30.9%)	271 (45.5%)	107 (18.0%)	29 (4.9%)	596	<0.001
**Mean Number of Colonoscopies Per Patient**	10.9 (IQR, 6-17; range, 2-24)	10.5 (IQR, 6-18; range, 2-22)	8.0 (IQR, 5-10; range, 2-15)	6.3 (IQR, 3-11; range, 2-11)	9.9 (IQR, 5-14; range, 2-24)	<0.001
**Mean Number of Adenomas Per Colonoscopy Procedure (MAP)**	0.32 (IQR, 0-3; range, 0-4)	0.61 (IQR, 0-5; range, 0-7)	0.82 (IQR, 0-5; range, 0-5)	0.44 (IQR, 0-1; range, 0-2)	0.54 (IQR, 0-5; range, 0-7)	<0.001
**Mean Number of Adenomas Per Positive Colonoscopy Procedure (MPP+)**	1.39 (IQR, 1-3; range, 1-4)	1.59 (IQR, 1-5; range, 1-7)	1.66 (IQR, 1-5; range, 1-5)	1.33 (IQR, 1-1; range, 1-2)	1.55 (IQR, 1-5; range, 1-7)	0.637
**Mean Number of Advanced Adenomas Per Colonoscopy Procedure**	0.01 (IQR, 0-0; range, 0-2)	0.03 (IQR, 0-1; range, 0-2)	0.04 (IQR, 0-1; range, 0-2)	0.02 (IQR, 0-0; range, 0-1)	0.02 (IQR, 0-2; range, 0-2)	0.391
**Mean Number of Adenomas Per Patient For Follow-Up Year**	0.31 (IQR, 0-1.86; range, 0-3.88)	0.90 (IQR, 0-4.08; range, 0-4.08)	0.84 (IQR, 0-2.50; range, 0-4.34)	0.56 (IQR, 0-0.91; range, 0-1.87)	0.66 (IQR, 0-4.08; range, 0-4.34)	<0.001
**Mean Number of Advanced Adenomas Per Patient For Follow-Up Year**	0.03 (IQR, 0-1.90; range, 0-1.90)	0.05 (IQR, 0-0.92; range, 0-0.92)	0.07 (IQR, 0-0.80; range, 0-0.80)	0.03 (IQR, 0-0.11; range, 0-0.11)	0.04 (IQR, 0-1.90; range, 0-1.90)	0.089
**CRC Count**	12 (22.2%)	5 (8.3%)	2 (6.1%)	0 (0%)	19 (11.7%)	0.002

Note that patients can present with more than one adenoma or advanced adenoma.

## Results

### Demographic characteristics of study participants

A total of 163 patients were enrolled in our cohort from 2013 to 2020, with visits and patient data spanning from 1997 to 2020 (**Table 1**): 33.1% carried a variant in *MLH1*, 36.8% in *MSH2/EPCAM*, 20.2% in *MSH6*, and 9.8% in *PMS2*. Overall, 58.3% were female, 79% were White, 4% Black, 4% Asian, 1% American Indian, and 12% were classified as other race. Regarding ethnicity, 14% identified themselves as Hispanic/Latino. 69% of patients in this cohort had previous history of cancer (survivors), with most survivors having CRC. Of all participants in the study, 29% reported a prior history of smoking and 12% were current smokers. Furthermore, 21% of participants self-reported that they were taking aspirin. Finally, a total of 7% of participants met criteria to be considered LTFU at the time of data analysis. Surgical history was also broken down by MMR gene (**Supplementary Table 1**) and described by survivorship status with most surgeries being right hemicolectomy procedures (**Supplementary Table 2**). We found that rates of right hemicolectomy were significantly higher among MLH1 and MSH2/EPCAM carriers and that survivors and patients with active cancer had a higher prevalence of colorectal surgeries, as expected (both, *P*<0.001).

### Outcomes

From a total of 761 clinic visits, 596 were colonoscopies while the remaining 165 visits included upper GI procedures and non-procedural visits. Among the colonoscopies, 25.3% were performed before 2013, and 74.7% from 2013 onwards. Patients had an average of 10 colonoscopies (IQR, 6-14; range, 2-24, **Table 2**), with an average age at first colonoscopy of 46 years (IQR, 37-55; range, 18-73) and an average age at subsequent follow-up of 53 years (IQR, 44-62; range, 18-82). At the first colonoscopy, 21.4% of the patients had at least one adenoma. Of these patients, 66.7% had tubular adenomas, 15.2% sessile serrated adenomas, and 18.2% advanced adenomas.

The MAP was calculated as 0.54 (IQR, 0-5; range, 0-7, **Table 2**). We observed statistically significant differences in the number of adenomas among the different MMR gene carriers (*P*<0.001) with *MSH6* carriers having the most adenomas (0.82; IQR, 0-5; range, 0-5) followed by *MSH2/EPCAM* (0.61; IQR, 0-5; range, 0-7), *PMS2* (0.44; IQR, 0-1; range, 0-2) and *MLH1* (0.32; IQR, 0-3; range, 0-4). The MPP+ was calculated as 1.55 (IQR, 1-5; range, 1-7) with no statistically significant difference between MMR carriers (*P*=0.637). The mean number of adenomas per patient for each year of follow-up was 0.66 (IQR, 0-4.08; range, 0-4.34) with *MSH2/EPCAM* carriers having the greatest average number of adenomas per year relative to other carriers (IQR, 0-4.08; range, 0-4.08; *P*<0.001). The mean number of advanced adenomas per procedure was 0.02 (IQR, 0-2; range, 0-2), though we found no statistically significant difference by MMR pathogenic variant carriers (*P=*0.391). The mean number of advanced adenomas per patient for each year of follow-up was 0.04 (IQR, 0-1.90; range, 0-1.90) with no statistically significant difference between MMR variant carriers (*P*=0.089). The mean interval between follow-up was 13.1 months (IQR, 0-59; range, 0-128), and the mean duration of follow-up in our cohort over the entire enrollment period was 8.7 years (IQR, 0-22, range, 0-22). The mean interval between colonoscopies was 13.9 months (IQR, 11-15; range, 0-128) and this was not significantly different among the MMR gene groups, thus reflecting previous historical surveillance recommendations. Moreover, we found no statistically significant difference in the mean intervals between colonoscopies by race or ethnicity (*P=*0.580, *P=*0.124, respectively).

A total of 19 patients were diagnosed with CRCs within our cohort for the total follow-up period. Nine patients were referred to our institution with the diagnosed cancer, and five were diagnosed at their first colonoscopy. Of the remaining patients, three were diagnosed on their second visit and two on subsequent visits. Moreover, two of the 19 patients displayed metachronous tumors that occurred within a year of their first tumor diagnosis. A total of three of 19 patients had *in-situ* (stage 0), four stage I, six stage II, and five stage III tumors. There was missing stage information for one patient. Twelve of these tumors (22.2%) were diagnosed in *MLH1* carriers, five (8.3%) from *MSH2/EPCAM*, and 2 (6.1%) from *MSH6* carriers (**Table 2**). For carriers who were not diagnosed with CRC in their initial visit, the average interval from last colonoscopy to diagnosis of CRC was 11.6 months (SD 6.5).

### Multivariate analyses

We conducted multivariate regression analyses to investigate the association between LS carrier profiles with likelihood of developing adenomas and CRC, controlling for ethnicity, race, gender, age, smoking status, and surgical history for the entire enrollment period. Compared to *MLH1* carriers, we found no statistically significant difference in the likelihood of developing adenomas between *MSH2/EPCAM, MSH6*, and *PMS2* (**Table 3**). As expected, participants ≥45 years old were more likely to develop adenomas compared to younger participants (OR 2.61, 95% CI 1.08-1.84, *P=*0.034). We found no statistically significant difference between different racial groups and the likelihood of developing adenomas. Furthermore, we found that, compared to previvors, participants with a prior history of cancer or active CRC were less likely to develop adenomas, even when controlling for age, gender, ethnicity, race, gene variant, and surgical history (OR 0.32, 95% CI 0.13-0.80, *P*=0.015). Surgical history was not associated with a statistically significant difference in the likelihood of adenoma development (*P=*0.868).

**Table 3:** Multivariate logistic regression for likelihood of adenoma development.

Factor	OR	95% CI	*P-*Value
MMR Variant			
MLH1	1	–	–
MSH2/EPCAM	0.97	0.50-1.89	0.923
MSH6	1.70	0.78-3.70	0.18
PMS2	1.16	0.32-4.20	0.816
Race			
White or Caucasian	1	–	–
Black or African American	–	–	–
Asian	0.49	0.06-4.30	0.519
American Indian	–	–	–
Other	–	–	–
Unknown	0.8	0.19-1.97	0.636
Ethnicity			
Non-Hispanic	1	–	–
Hispanic	1.47	0.91-2.39	0.118
Sex			
Male	1	–	–
Female	1.02	0.57-1.84	0.937
Age			
Age <45	1	–	–
Age ≥45	2.61	1.08-1.84	0.034
Smoking Status			
No documented smoking history	1	–	–
Prior or current smoker	0.8	0.52-1.23	0.304
Status			
Previvor	1	–	–
Survivor and Active Cancer	0.32	0.13-0.80	0.015
Surgical History			
No Surgical History	1	–	–
Surgical History	0.95	0.51-1.75	0.868

OR, Odds Ratio; CI, Confidence Intervals.

We assessed the association between gene variation and likelihood of developing an advanced adenoma (**Table 4**) and CRC development (**Table 5**) in which we controlled for the same covariates as in our prior analysis. While we controlled for both race and ethnicity in measuring the association between gene variant and advanced adenomas, this regression was only adjusted by race as, when controlling for race and ethnicity, the logistic regression did not converge. However, we did not find any statistically significant association between any of our measured variables and the likelihood of developing an advanced adenoma or CRC, likely due to the relatively sparse number of advanced adenomas and CRC cases within our cohort.

**Table 4:** Multivariate logistic regression for likelihood of advanced adenoma development OR, Odds Ratio; CI, Confidence Intervals.

Factor	OR	95% CI	*P-*Value
MMR Variant			
MLH1	1	–	–
MSH2/EPCAM	0.72	0.16-3.16	0.659
MSH6	0.84	0.13-5.41	0.852
PMS2	1.77	0.16-19.43	0.639
Race			
White or Caucasian	1	–	–
Black or African American	–	–	–
Asian	–	–	–
American Indian	–	–	–
Unknown Race/Ethnicity	–	–	–
Other	1.02	0.155-6.79	0.985
Ethnicity			
Non-Hispanic Ethnicity	1	–	–
Hispanic Ethnicity	1.56	0.57-4.28	0.384
Sex			
Male	1	–	–
Female	0.96	0.25-3.74	0.951
Age			
Age <45	1	–	–
Age ≥45	0.78	0.12-4.90	0.788
Smoking Status			
No documented smoking history	1	–	–
Prior or current smoker	1.76	0.52-6.01	0.366
Status			
Previvor	1	–	–
Survivor and Active Cancer	0.52	0.08-3.57	0.505
Surgical History			
No Surgical History	1	–	–
Surgical History	1.04	0.24-4.47	0.961

**Table 5:** Multivariate logistic regression for likelihood of CRC.

Factor	OR	95% CI	*P-*Value
MMR Variant			
MLH1	1	–	–
MSH2/EPCAM	0.74	0.11-5.23	0.764
MSH6	0.84	0.07-9.46	0.888
PMS2	–	–	–
Race			
White or Caucasian	1	–	–
Black or African American	–	–	–
Asian	4.39	0.28-75.5	0.287
American Indian	–	–	–
Other	1.41	0.15-15.52	0.728
Unknown	–	–	–
Sex			
Male	1	–	–
Female	0.28	0.03-2.95	0.288
Age			
Age <45	1	–	–
Age ≥45	0.67	0.08-5.53	0.709
Smoking Status			
No documented smoking history	1	–	–
Prior or current smoker	0.81	0.25-2.62	0.728
Status			
Previvor	1	–	–
Survivor and Active Cancer	–	–	–
Surgical History			
No Surgical History	1	–	–
Surgical History	1.2	0.20-7.31	0.842

OR, Odds Ratio; CI, Confidence Intervals.

### Cumulative incidence analysis

**Figure 1:**
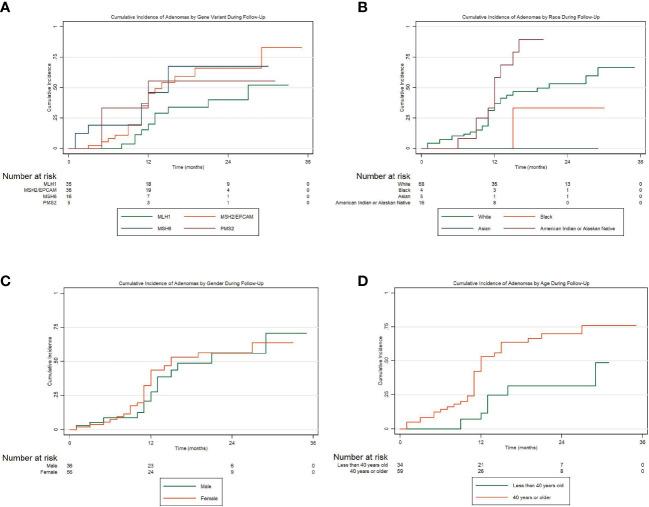
**(A)** Cumulative incidence of all colorectal adenomas by MMR gene variation over a period of 36 months of follow-up; **(B)** Cumulative incidence of all adenomas stratified Hispanic ethnicity; **(C)** Cumulative incidence of all adenomas stratified by gender; **(D)** Cumulative incidence of all adenomas stratified by age.

**Figure 2:**
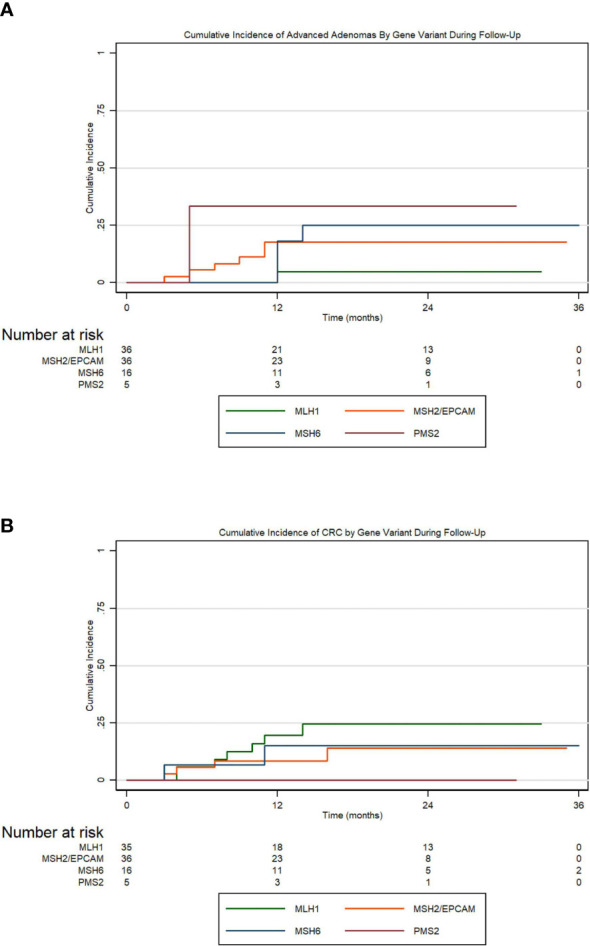
**(A)** Cumulative incidence of advanced adenomas by MMR variation over a period of 36 months of follow-up; **(B)** Cumulative incidence of colorectal cancer by MMR gene variation.

**Figure 3:**
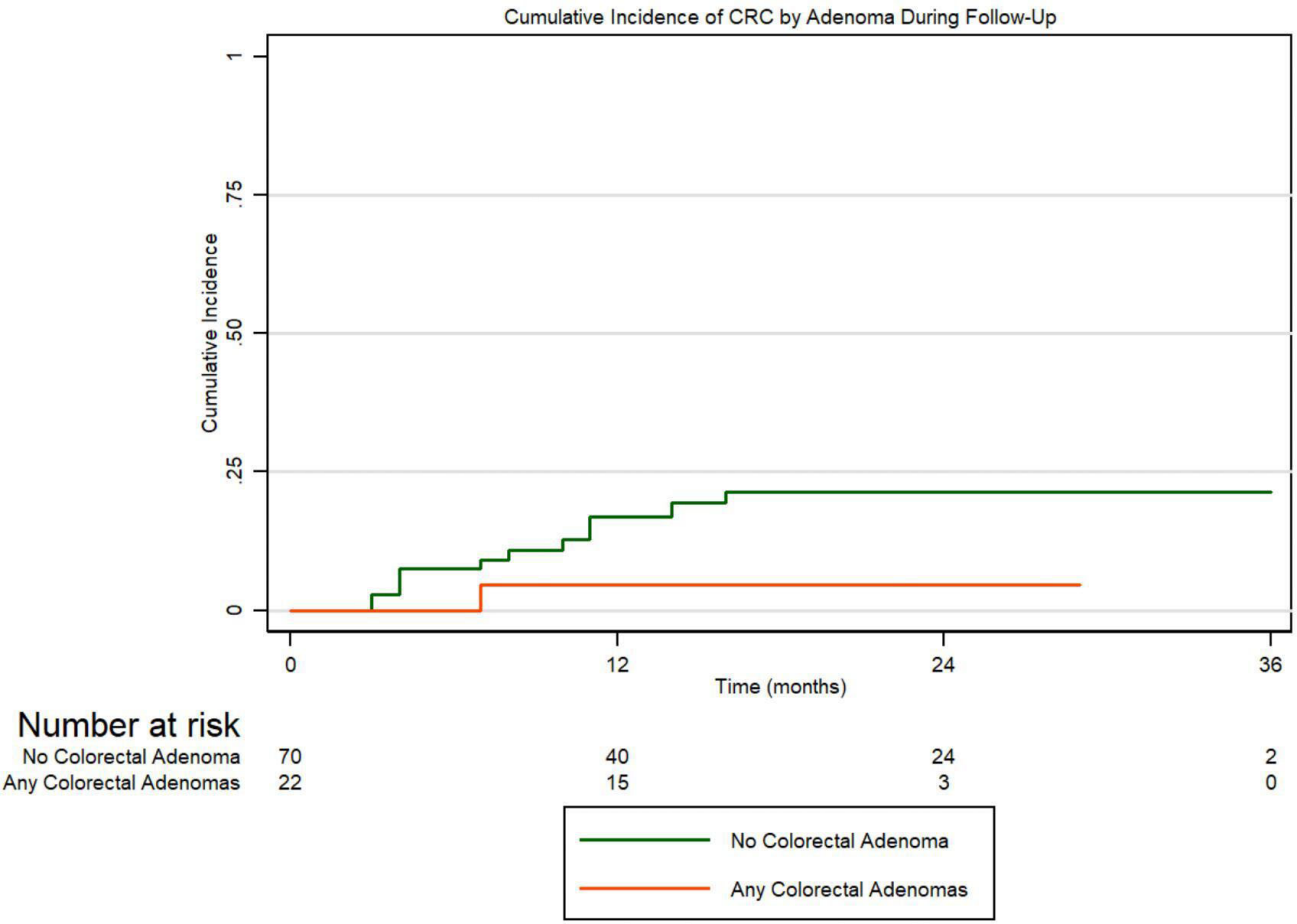
Cumulative incidence of colorectal adenoma progression to CRC.

To assess the incidences for adenomas, advanced adenomas, and CRC within the first 36 months following enrollment, we conducted Kaplan-Meier cumulative incidence analyses and tested for significance using Cox proportional hazard regression. **Figure 1A** presents the cumulative incidence of all adenomas by MMR gene variation for 36 months. Carriers were considered at-risk from time of enrollment to first adenoma development if they did not have an adenoma at their initial visit. We conducted Cox proportional hazard regression for the risk of developing adenomas by MMR gene carriers. Compared to *MLH1* carriers, we found that *MSH2/EPCAM* carriers were more likely to develop adenomas (HR 2.17, 95% CI 1.01-4.66, *P*=0.047). **Figure 1B** presents the cumulative incidence of all adenomas by ethnicity in the same period. Overall, there is a non-statistically significant trend for Hispanics having a higher cumulative incidence of all adenomas and for developing adenomas earlier when compared to non-Hispanics patients (*P=*0.073). **Figure 1C** represents the cumulative incidence of all adenomas by gender displaying no significant differences in the incidence of adenomas when compared males to females in our cohort (*P*=0.473). **Figure 1D** represents the cumulative incidence of all adenomas stratified by age at enrollment. Patients 45 years or older had a significantly higher incidence of adenoma development than patients under 45 (HR 2.99, 95% CI 1.38-6.51, *P=*0.006). Regarding advanced adenomas, **Figure 2A** presents the cumulative incidence by MMR gene during the period of follow-up. Patients were considered at-risk from time of enrollment to the development of first advanced adenoma captured under surveillance if they did not have an advanced adenoma or CRC diagnosed at their initial visit. The Kaplan-Meier curve demonstrates a pattern where *PMS2* carriers appeared to have the greatest cumulative incidence of advanced adenomas in our cohort while the *MSH2/EPCAM* carriers had an earlier cumulative incidence for these adenomas. Importantly, however, we found no statistically significant differences in the incidence of advanced adenoma between *MLH1* and *MSH2/EPCAM, MSH6*, and *PMS2* (*P*=0.105, *P*=0.111, *P*=0.093, respectively). **Figure 2B** represents the cumulative incidence of CRC by genetic variant. Patients were considered at-risk for CRC from study enrollment to CRC development if they were not diagnosed with CRC at their initial visit. We did not observe differences between *MLH1* and other carrier subgroups (*P*=0.198). Finally, **Figure 3** represents the cumulative incidence of colorectal adenoma progression to CRC in our cohort for this same period. We did not observe an association between adenoma development and CRCs (*P*=0.160).

## Discussion

Despite the well-known relationship between the MMR variants and CRC risk that has led to the implementation of intense screening programs through colonoscopy, LS carriers continue to be diagnosed with CRC. In this study, we analyzed a demographically diverse cohort of LS patients from a single institution tertiary care center engaged in LS screening through several decades and reported associations between carrier characteristics with outcome development. We performed logistic regression analyses to assess the likelihood of these outcomes for MMR gene, race and ethnicity, sex, age, smoking status, surgical history, and cancer status across all collected patient data.

We also performed cumulative incidence analysis of adenomas and advanced neoplasia (i.e., advanced adenomas and CRCs) for these carriers during a 36-month follow-up period. To visually represent the cumulative incidence for adenomas and advanced neoplasia during this period, we generated Kaplan-Meier curves and tested their significance using Cox proportional regression.

Current guidelines by the National Comprehensive Cancer Network (NCCN) suggest earlier and more frequent colonoscopies in patients with *MLH1, MSH2*, and *EPCAM* variants. However, there is not yet a consensus on interval screening for this patient population. The British Society of Gastroenterology/Association of Coloproctology of Great Britain and Ireland (BSG/ACPBGI) guidelines has recommended interval screenings every 2 years for all patients with known MMR variants (12). In contrast, the American College of Gastroenterology’s guidelines recommend yearly screenings in patients with known MMR variants but every 2 years for those who might be at risk or affected by LS (13). Here, we report a statistically significant greater and an earlier incidence of adenomas for *MSH2*/*EPCAM* carriers compared to *MLH1* over a 36-month follow-up period. However, we did not observe statistically significant associations between adenomas and MMR carriers. These findings support the NCCN’s recommendations to start screening colonoscopies earlier in *MSH2* carriers, as they may be more likely to develop adenomas earlier compared to other LS carriers. However, the NCCN’s guidelines propose time wide screening intervals (i.e., 1-3 years) rather than individualized guidance, which permits significant variability in colonoscopy frequency by individual providers and centers’ preferences. Analysis from the Prospective Lynch Syndrome Database (PLSD) showed that colonoscopies more frequent than once every three years did not necessarily reduce the incidence of CRC or outcomes upon diagnosis (4). In addition, analysis from this database showed that the removal of adenomas in a LS patient cohort did not decrease the incidence of CRC. In practical terms, these intervals are often adjusted to reflect the individual occurrence of adenomas in each LS carrier. Our adenoma incidence findings of *MSH2/EPCAM* carriers are in line with others previously documented in the literature; however, as stated, we did not observe a statistically significant difference in prevalence of adenoma between MMR variants on multivariate analysis when the entire follow-up for the cohort was considered. This discrepancy may be attributable to size of the cohort, the increased surveillance for the participants in our study relative to the general LS population and the influence of surgical history among survivors. As our Kaplan-Meier curves measured incidence for the first 36-months following the initial appointment, this discrepancy may also suggest that the likelihood of adenoma development for *MSH2* and *EPCAM* may be attenuated following consistent surveillance. These suggestive findings provide additional information on pre-cancer incidence that can help to tailor screening intervals by MMR genotype. Based on expert opinion, LS patients who benefit from annual rather than biennial or triennial coloscopies are those with a prior history of CRC or adenomas, carriers greater than 40 years of age and males (11). Although our findings show that male carriers have a higher incidence of adenomas, this was not determined to be statistically significant on Cox proportional regression or multivariate analysis. Our findings demonstrated a significantly increased likelihood of adenoma development within our cohort for participants 45 years or older. Further, we found that participants with an active or prior history of cancer were less likely to develop subsequent adenomas on multivariate analysis. While we found a statistically significant difference in the rate of prior colorectal surgeries by MMR gene carrier, surgical history was not associated with adenoma development on multivariate analysis. Therefore, more studies are needed to validate these recommendations and to find more data-driven associations that can improve the level of evidence behind current guidelines to match the clinical reality.

Based on our results, we propose that other patient demographics, specifically race and ethnicity, should be considered in estimating the risk of developing CRC for LS patients. Although LS carriers carry significant lifetime risk for the development of CRC regardless of ethnicity and race, patients belonging to racial and ethnic minorities in the United States are less likely to receive genetic evaluation for inherited CRC syndromes and may not receive sufficient screening following diagnosis (15). This discrepancy has been correlated to a lack of patient education on adequate screening modalities leading to stigmatization of screening practice, the potential effects of oncologists’ implicit biases in patient-physician interactions, and the insurance disparities between Hispanic and non-Hispanic populations (16–18). In contrast to the literature, we showed no significant difference in interval screening between Hispanics and non-Hispanics in our cohort. This observation may be attributable to the consistency in screening practices within our cohort referred to a tertiary care center or may otherwise indicate that LS carriers enrolled in our study may be less likely to face those barriers to care. Furthermore, while Hispanic carriers had a greater and earlier incidence of adenomas compared to non-Hispanic patients, this difference was also not determined to be significant on Cox proportional hazard or multivariate analysis. To our knowledge, this is the first analysis that specifically controlled for ethnicity when evaluating cumulative adenoma incidence over a follow-up period in a LS population. While prior investigations have shown an increased mortality rate within CRC for these populations, to our knowledge, no study has specifically investigated the time to adenoma development for this patient cohort (19). Given that Hispanic patients often develop adenomas and CRC at a younger age, have a greater incidence of *MLH1* and *PMS2* variants, and are less likely to receive appropriate screening, these patients represent a vulnerable population that may require further analysis for evidence-based screening and management guidelines (14). Therefore, our findings highlight the need for further investigation into potential disparities in screening and effective interventions for Hispanic LS carriers.

We acknowledge several limitations of our study. While the enrolled patient cohort was demographically diverse, we recognize that the population analyzed may have limited generalizability to LS patients worldwide. Second, we did not establish a significant difference in patient outcomes based on MMR profiles or ethnicity. Because patients enrolled in our study underwent regular screenings, the outcomes for the LS patient cohort may not reflect the outcomes in an unmonitored patient population. Third, our follow-up period was limited to 36 months post-enrollment, so we did not generate differences in follow-up among participants that would have been difficult for us to control. We continue to follow up the outcomes in our patient cohort, but the generalizability of our current results reported here is limited within this 36-month period. Therefore, longer monitoring may reveal more robust and generalizable clinical outcomes. Fourth, we did not collect specific information on the prevalence of other LS-related cancers; thus, we did not capture the risk for gynecological or other GI tumors. Fifth, we were unable to account for the potential influence of the significant advancements in endoscopic technologies from the earliest available patient data (in 1997) to present and did not capture Key Performance Indicators (KPI) to approximate for the quality of colonoscopies performed for this cohort. Sixth, we described the number of patients who reported taking aspirin, but we did not systematically collect data on aspirin usage such as dosing throughout the study and did not therefore account for this in our multivariate analysis. Finally, we did not exclude patients with a history of colorectal surgery, which limits the potential for adenoma and CRC development in a subgroup of LS survivors.

Our study demonstrates several strengths. The patient cohort represented patients from various age groups, which permits greater generalizability of our results to a larger patient cohort. Furthermore, we captured the frequency and onset of adenomas for a vulnerable patient population and further stratified by ethnicity. We were also able to follow the patient cohort over an extended period with minimal loss to follow-up, which allowed us to thoroughly monitor the incidence of adenoma development. As the database continues to be updated, the correlation between demographic information and potential outcomes of interest may improve. LS patients in our database were collected and analyzed for adenoma incidence based on demographic information (such as race and gender) and MMR carrier status. We further investigated the degree to which these characteristics were associated with advanced adenomas and the development of CRC. The maintenance of a robust database of LS patients with a variety of heredity MMR variants is necessary for the creation of biobanks with prospective tissue collection, providing greater insights into the carcinogenesis of LS from a multi-specialty perspective. This database will allow providers to more precisely tailor treatment plans to individual patients based on constitutional variant status and patient characteristics. Finally, while we observed that Hispanic carriers experienced a greater cumulative incidence of adenomas, we did not observe a statistically significant difference in the cumulative incidence of adenomas by Hispanic ethnicity for this cohort. These results highlight the need for a well-maintained database of LS patients to facilitate proper surveillance and appropriate intervention management for Hispanic LS carriers, as they are not necessarily more prone to the development of adenomas compared to non-Hispanic carriers. Therefore, given the deficiencies in the screening infrastructure documented extensively in the medical literature for Hispanic patients and racial minorities, further studies are warranted to determine the efficacy of established LS guidelines for demographically diverse patient populations and modify them accordingly.

## Data availability statement

The raw data supporting the conclusions of this article will be made available by the authors, without undue reservation.

## Ethics statement

The studies involving human participants were reviewed and approved by MD Anderson Cancer Center IRB. The patients/participants provided their written informed consent to participate in this study.

## Author contributions

EV contributed to the conception and design of the study. PL, MR-B, ST, MT, YY, DW, VS, and EV were involved in patient enrollment. JM, MM, PL, MR-B, ST, MT, YY, and EV identified the study subjects and provided clinical information. LR-U, JK, KE, and CB organized and maintained the database. GDC and LR-U performed statistical analysis. GDC, LR-U, DG, and EV wrote the manuscript and provided revisions of the manuscript. EV provided critical review of the manuscript. All authors contributed to the article and approved the submitted version.
