# Supplementary material for: Colorectal surveillance outcomes from an institutional longitudinal cohort of lynch syndrome carriers
Source: Front Oncol. 2023 Apr 24;13:1146825. doi: 10.3389/fonc.2023.1146825 (PMC10164917; doi:10.3389/fonc.2023.1146825)
Supplement: Supplementary file 1 [file Table_1.pdf]

Supplementary Table 1. History of Colorectal and Small Bowel Surgeries Based on MMR Gene. Note that patients can have more than one type of surgery**.**

Surgery	*MLH1*	*MSH2/EPCAM*	*MSH6*	*PMS2*	*P-* Value
Right hemicolectomy	17 (56.7%)	12 (46.2%)	6 (60.0%)	2 (33.3%)	<0.001
Total Colectomy with Ileostomy	0 (0%)	2 (7.7%)	0 (0%)	0 (0%)	<0.001
Total Colectomy with Ileorectal	4 (13.3%)	0 (0%)	0 (0%)	0 (0%)	<0.001
Left hemicolectomy	3 (10.0%)	1 (3.8%)	0 (0%)	1 (16.7%)	<0.001
Sigmoid colon resection	1 (3.3%)	4 (15.4%)	0 (0%)	0 (0%)	<0.001
Other colorectal surgeries	4 (13.3%)	6 (23.1%)	3 (30.0%)	3 (50.0%)	<0.001
Small bowel surgeries	1 (3.3%)	1 (3.8%)	1 (1.0%)	0 (0%)	0.002
