# Supplementary material for: Colorectal surveillance outcomes from an institutional longitudinal cohort of lynch syndrome carriers
Source: Front Oncol. 2023 Apr 24;13:1146825. doi: 10.3389/fonc.2023.1146825 (PMC10164917; doi:10.3389/fonc.2023.1146825)
Supplement: Supplementary file 2 [file Table_2.pdf]

Supplementary Table 2. History of Colorectal and Small Bowel Surgeries Based on Patient Status at Time of Enrollment (Previvor vs. Active Cancer and Survivor). Note that patients can have more than one type of surgery**.**

Surgery	Previvor N=50	Active Cancer/Survivor N=112	*P* -Value
No Surgical History	49 (98.0%)	43 (38.4%)	<0.001
Right hemicolectomy	0 (0%)	35 (31.3%)	<0.001
Total Colectomy with Ileostomy	0 (0%)	1 (0.9%)	0.001
Total Colectomy with Ileorectal Anastomosis	0 (0%)	4 (3.6%)	0.003
Left hemicolectomy	0 (0%)	6 (5.4%)	0.001
Sigmoid colon resection	0 (0%)	5 (4.5%)	<0.001
Other colorectal surgeries	0 (0%)	16 (14.3%)	0.536
Small bowel surgeries	1 (2.0%)	2 (1.8%)	0.134
